# Effects of perioperative clinical hypnosis on heart rate variability in patients undergoing oncologic surgery: secondary outcomes of a randomized controlled trial

**DOI:** 10.3389/fpain.2024.1354015

**Published:** 2024-03-08

**Authors:** Muhammad Abid Azam, Aliza Z. Weinrib, P. Maxwell Slepian, Brittany N. Rosenbloom, Anna Waisman, Hance Clarke, Joel Katz

**Affiliations:** ^1^ Department of Psychology, York University, Toronto, ON, Canada; ^2^ Department of Anesthesia and Pain Management, Toronto General Hospital, Toronto, ON, Canada; ^3^ Department of Anesthesiology and Pain Medicine, University of Toronto, Toronto ON, Canada; ^4^ Child Health Evaluative Sciences, The Hospital for Sick Children, Toronto, ON, Canada

**Keywords:** clinical hypnosis, oncologic surgery, postoperative opioid use, postoperative pain, high frequency heart rate variability

## Abstract

**Introduction:**

Clinical hypnosis has been proposed for post-surgical pain management for its potential vagal-mediated anti-inflammatory properties. Evidence is needed to understand its effectiveness for post-surgical recovery. Iin this secondary outcome study, it was hypothesized that surgical oncology patients randomized to receive perioperative clinical hypnosis (CH) would demonstrate greater heart-rate variability (HRV) during rest and relaxation at a 1-month post-surgery assessment compared to a treatment-as-usual group (TAU).

**Methods:**

After REB approval, trial registration and informed consent, 92 participants were randomized to receive CH (*n* = 45) or TAU (*n* = 47). CH participants received a CH session before surgery and during post-surgical in-hospital stay HRV was assessed during rest (5 min) and relaxation (10 min) before and 1-month after surgery. Pain intensity was obtained using a 0–10 numeric rating scale pre and post 1-week and 1-month post surgery.

**Results:**

One month after surgery, HRV was significantly higher in CH group (*n* = 29) during rest and relaxation (both *p* < 0.05, *d* = 0.73) than TAU group (*n* = 28). By contrast, rest and relaxation HRV decreased from pre- to 1-month post-surgery for the TAU (both *p* < 0.001, *d* > 0.48) but not the CH group. Pain intensity increased from pre-surgery to 1-week post-surgery (*p* < 0.001, *d* = 0.50), and decreased from 1-week to 1-month post-surgery (*p* = 0.005, *d* = 0.21) for all participants.

**Discussion:**

The results suggest that hypnosis prevents the deleterious effects of surgery on HRV by preserving pre-operative vagal activity. These findings underscore the potential of clinical hypnosis in mitigating the adverse effects of surgery on autonomic function and may have significant implications for enhancing post-surgical recovery and pain management strategies.

**Clinical Trial Registration:**

ClinicalTrials.gov, identifier (NCT03730350).

## Introduction

Hypnosis is one of the oldest treatments for the management of pain (1). Over the past twenty years, there has been renewed interest in the efficacy of clinical hypnosis as an opioid-sparing adjunct for managing acute and chronic pain. As a result of this body of evidence, the American Psychological Association has recommended that clinical hypnosis be included as part of standard care for pain relief (2, 3).

Hypnosis induces a temporary state of consciousness characterized by inner focus and deep relaxation, which is theorized to enhance receptivity to suggestions to alter subconscious physiological functions and symptoms such as pain, blood pressure, and gut motility (4). When providing clinical hypnosis to patients undergoing surgery, suggestions for positive expectations, increased coping abilities, and induction of relaxation are used to influence post-surgical outcomes (3), such as post-surgical pain intensity. Meta-analyses show that surgical patients who receive hypnosis have better outcomes (e.g., less pain intensity, better mood, and less medication use) than those who receive treatment-as-usual (5, 6). Given these promising findings, investigators have called for studies to evaluate clinically relevant biomarkers that can yield insight into the mechanisms underlying the effects of hypnosis for post-surgical recovery (7).

Recent evidence suggests the effects of hypnosis are mediated by the parasympathetic nervous system (PNS) potentially through cardiac vagal nerve activity (8). PNS functions are associated with maintaining key body functions, including the control of inflammation. PNS activity is subdued in the aftermath of major surgery (8–10), a period characterized by heightened inflammatory activity due to surgery-related tissue damage (9, 11). Prolonged weakening of the PNS may leave the body vulnerable to the adverse effects of excess inflammation, including sepsis and post-surgical pain (12).

PNS function can be measured by heart rate variability (HRV), a clinical biomarker of vagal heart rate modulation that is inversely associated with acute pain (13) and persistent pain disorders (14, 15). Moreover, high levels of inflammation are inversely related to HRV (16). These relationships are explained by a vagal anti-inflammatory reflex (17, 18), which is triggered by the detection of cytokines that initiate a cascade of cholinergic processes to inhibit inflammation (19). Putatively, a robust PNS and vagal anti-inflammatory reflex is signified by elevated HRV, whereas diminished HRV levels would signal a dysfunctional PNS.

Clinical hypnosis exerts a positive influence on vagal HR modulation (20, 21) and ameliorates sympathetic stress activity (22). To our knowledge, the effects of clinical hypnosis on HRV has not been studied in the oncology surgical setting, where preventive, opioid-sparing pain management interventions are sorely needed (7). The activation of the vagal nerve might modulate the analgesic effects of opioids. This suggests that vagal nerve stimulation could either enhance the effectiveness of opioids, allowing for lower doses to be used, or activate similar pain-relieving pathways in the brain, thus providing a non-opioid method for pain relief (23).

This randomized controlled trial (RCT) examined the effects of a pre- and post-operative clinical hypnosis intervention on cardiac vagal activity using HRV assessments, a secondary outcome of the RCT. The main RCT study found that participants who received hypnosis required significantly fewer opioids postoperatively compared to those who received standard care alone. Additionally, hypnosis was associated with a protective effect against the increase in pain catastrophizing scores one week after surgery. These findings suggest that clinical hypnosis can be a valuable tool in the perioperative management of pain, potentially reducing the need for opioid analgesics and mitigating negative psychological responses to pain (24).

In this present, secondary study, we hypothesized that participants randomized to the clinical hypnosis treatment group would demonstrate greater HRV during rest and relaxation at the 1-month post-surgery assessment compared to the treatment-as-usual group. We also hypothesized that the treatment-as-usual group, but not the clinical hypnosis group, would exhibit lower HRV during rest and relaxation at the 1-month post-surgery assessment compared to the corresponding pre-surgery assessment.

## Methods

### Study design

This is a secondary outcome study of a single-center, stratified, randomized-controlled, parallel-group trial conducted at the Toronto General Hospital's Transitional Pain Service (TPS; Toronto, Canada) (25). This study was designed according to the 2010 CONSORT statement (26), reviewed and approved by the University Health Network Research Ethics Board (certificate #: 17-5441) as well as the Human Participants Review Committee at York University (certificate #: e2019-031), and registered with ClinicalTrials.gov (registration #: NCT03730350) prior to recruitment of any participants. Data are not available to other researchers, but analytic methods and study materials are available upon request.

#### Participants

Eligible participants were all adults aged 18 years or older, scheduled for a surgical oncology procedure (e.g., thoracic, gastrointestinal, gynecologic, urologic, head and neck, breast cancer, etc.) at the Toronto General Hospital. Exclusion criteria included patients with limited comprehension of English who would not be able to understand the verbal instructions for clinical hypnosis, cognitive deficits due to dementia or other causes that would limit comprehension, or a history of serious mental illness (e.g., schizophrenia, dissociative identity disorder, PTSD with dissociation) for which hypnosis is contraindicated.

#### Sample size estimation

The sample size (*N* = 92) estimate was based on a power analysis of daily morphine equivalents in milligrams, post-surgery, which was the primary outcome of the main RCT (24). The present study's sample size was deemed suitable for analyses, as according to Quintana (27), samples between *N* = 61 to *N* = 233 are sufficient to achieve 80% power in detecting moderate to large effect sizes in HRV.

#### Randomization

##### Random sequence generation

A study co-investigator with no clinical involvement in the trial created a computer-generated randomization schedule (www.randomization.com) stratified by patient's current and/or past use of opioid medications or lack thereof (opioid-experienced vs. opioid naïve) with a 1:1 allocation using random block sizes of ten to ensure close balance of the numbers of each group at any time during the trial. Participants were randomly assigned following blocked randomization procedures to one of two intervention arms, clinical hypnosis or treatment-as-usual.

##### Allocation concealment

To ensure that the person randomizing participants to treatment condition would not be aware of the next treatment allocation, a study coordinator inserted colored cards containing the intervention assignment details inside sealed, opaque envelopes that were consecutively numbered with participant study ID numbers. The allocation sequence was concealed from the coordinator obtaining informed consent from participants as well as the outcome assessors, and the envelopes were opened and read only after participants had completed all pre-surgery assessments and it was time to allocate to intervention. Randomization took place at the end of the pre-surgery assessment (i.e., after measurement of pre-surgical HRV), when the outcome assessor and/or clinician opened the next consecutively numbered sealed envelope corresponding to the participant's study ID and informed the participant of their group assignment.

### Interventions

#### Clinical hypnosis intervention

Participants randomized to the clinical hypnosis arm of the study received standard perioperative care along with an adjunct perioperative clinical hypnosis intervention. One in-person session of clinical hypnosis was provided approximately 1–2 weeks prior to the day of surgery, and one in-person session of clinical hypnosis was provided in-hospital 1–3 days after the day of surgery (Table 2). The scripts for all hypnosis sessions were developed by the TPS pain psychologist and study co-investigator (AZW), based on the clinical literature (4) and manualized for use in this study. The hypnosis scripts were ACT-informed and made use of ACT principles (e.g., acceptance of acute pain) as well as direct and indirect suggestions for a reduction in pain intensity. The TPS psychologist and psychology trainees who provided the hypnosis intervention were trained and certified in clinical hypnosis by the Canadian Society of Clinical Hypnosis—Ontario Division.

##### Pre-surgery hypnosis

All participants in the treatment group provided informed consent to clinical hypnosis. The pre-surgery clinical hypnosis session was 20–25 min in duration and was aimed at preparing the participant for surgery by reducing anxiety and introducing relaxation and self-soothing strategies to be used before and after surgery for reducing stress and improving pain coping. Prior to the session, the clinician provided information on clinical hypnosis, including the use of hypnosis in the medical context and an overview of the pre-surgery hypnosis session. The participant was asked to think of a “special place” (e.g., a peaceful cottage on a lake, real or imagined) that is particularly relaxing for that individual to incorporate into the session. The pre-surgery hypnosis session included the following steps: (1) hypnotic induction with slow, deep breathing; (2) hypnotic deepening using progressive relaxation with suggestions of warmth spreading through the body; (3) suggestions of mental imagery and pleasurable sensory experiences in a “special place” of participant's choosing; (4) suggestions of positive imagery for the participant's experiences leading up to, during, and after surgery; (5) suggestions for the participant to engage in self-hypnosis before surgery and during surgical recovery; and (6) alerting with counting. While providing hypnosis, the clinician observed the participant and adapted delivery of instructions according to observed bodily cues of relaxation (e.g., facial muscles relaxing, slow breathing rhythm). Participants were given the option to lie prone on a hospital bed, or be seated, for the duration of the session. Participants were asked to keep their eyes closed during the session, to limit visual distractions and enhance inner focus. Sessions were provided in a private room with doors closed to reduce distracting noise. Participants were also provided with a recording of the pre-surgery hypnosis script to use at home, with instructions that they listen to it on the two days prior to their scheduled surgery.

##### Post-surgery hypnosis

Following surgery, a clinician from the TPS pain psychology team visited the participant in hospital on post-operative day one or whenever the patient was sufficiently alert. The post-surgical hypnosis session occurred during the inpatient stay in the patient's hospital room prior to hospital discharge to guide them through a clinical hypnosis session at the bedside, 15–20 min in duration, targeted at increasing comfort and pain relief. For this session, participants were given the option to incorporate their chosen “special place” of comfort and relaxation, or to be guided in a hypnosis session of having a “trip to the beach”. In addition, participants were given the option to be alerted and re-awakened at the end of this session or be left to drift off to sleep if desired. The post-surgery hypnosis session included the following steps: (1) hypnotic induction with slow, deep breathing; (2) suggestions for minimizing impact of hospital room noise, (3) hypnotic deepening using progressive relaxation with suggestions of warmth; (4) suggestions of mental imagery and pleasurable sensory experiences in a “special place” of participant's choosing, or for a “trip to the beach”; (5) suggestions for sensory substitution, reduction of pain intensity, and reduction of pain unpleasantness; (6) suggestions for participant to engage in self-hypnosis as needed during recovery; and (7) alerting with counting, or leaving participant to drift off to sleep.

Audio hypnosis tracks developed by the TPS psychologist were provided for independent use after surgery, with daily practice recommended. These audio tracks were accessible on the study's private YouTube webpage. If participants were not able to use a personal device to access audio hypnosis tracks during their post-surgical recovery, they were provided an MP3 audio player to use for the duration of their hospital stay. Each of these tracks was 20–25 min in duration, and aimed at promoting pain relief, reducing distress and anxiety, and facilitating sleep. Each track contained two endings which the participant could choose between—one to alert and awaken the patient at the end of the track to continue with their day, and one that did not alert the patient and facilitated drifting off to sleep if so desired. Patients were provided with daily tracking logs to track how frequently they practiced hypnosis before and after surgery.

#### Treatment-as-usual

Participants randomized to the treatment-as-usual arm of the study received standard perioperative care. Participants in this study arm were offered to undergo a session of in-person hypnosis at the one-month assessment, after all of the physiological and questionnaire measures had been completed. Hypnosis audio tracks were made available to participants randomized to the treatment-as-usual arm at the one-month appointment after completion of all assessments.

#### Blinding

Study personnel, including coordinators, outcome assessors, and clinicians providing the hypnosis interventions were not blinded to treatment condition. Coordinators corresponded with participants to schedule post-surgery assessments, at which time they provided them with relevant questionnaires based on the intervention arm to which they had been randomized, and, in the case of the treatment-as-usual participants, to offer them an in-person session of clinical hypnosis after completion of the one-month assessment. Clinicians were required to be aware of the intervention arm to which participants had been allocated to determine whether to provide a pre-surgery hypnosis session after the pre-surgery assessment was completed. In some cases, clinicians who provided the clinical hypnosis served the dual role as outcome assessors conducting the physiological assessments. Steps were taken to minimize potential bias in outcome assessors, such as randomizing to intervention arms (i.e., opening sealed envelopes) only after pre-surgery assessments were completed. Finally, participants were not blinded to study intervention, partly due to the lack of adequate “sham” or placebo hypnosis procedures to serve as appropriate controls for clinical hypnosis (28).

### Outcomes

#### Heart rate variability

Electrocardiogram (ECG) recordings were collected using MindWare Impedance Cardiograph acquisition system (Ohio, United States), and used to analyze HRV; the beat-to-beat variation in either heart rate or the inter-beat intervals used to index parasympathetic nervous system activity. Using the MindWare equipment, we applied 3 adhesive electrodes to the participants' right collarbone (−) and the lower left and right ribs (+ and ground), or alternatively their wrists (+ and −) and ankle (ground). MindWare BioLab and HRV software were used to calculate high-frequency HRV (HF-HRV) that is widely used to indirectly estimate vagal HR modulation (29). The HF component of HRV is quantified in the frequency range of 0.15 to 0.40 Hz, reflecting respiratory sinus arrhythmia and parasympathetic modulation of the heart rate. The process involves the following steps: (1) Preprocessing: The raw electrocardiogram (ECG) signal is processed to detect R-waves, from which the IBIs (or RR intervals) are derived. The software applies filters to remove artifacts and arrhythmic beats, ensuring that only accurate, normal-to-normal (NN) intervals are analyzed. (2) Spectral Analysis: Using the cleaned IBI series, the software then performs spectral analysis via FFT or autoregressive methods. This analysis decomposes the IBI series into its frequency components, allowing for the quantification of power (measured in ms^2) within specified frequency bands, including the HF band. (3) HF HRV Calculation: The power within the 0.15 to 0.40 Hz frequency range is calculated and reported as HF HRV. Normative values for HF-HRV (ms/Hz) are as follows: 94.6, with an interquartile range from 54.6 to 150 (30). The values are recommended to undergo log-transformation, to normalize the data distribution and reduce skewness. These values represent the variance (or power) of heart rate oscillations associated with the respiratory cycle and are considered a marker of vagal tone or parasympathetic nervous system activity.

Assessments for participants in both the clinical hypnosis and the treatment-as-usual groups were conducted in a controlled environment — a quiet, closed lab room. These assessments were carried out during two distinct phases to ensure baseline comparability between groups. The first phase involved a 5 min rest period where participants were seated comfortably with their eyes closed but not subjected to any form of hypnotic or relaxation intervention. The second phase, applied only to the clinical hypnosis group, involved a 10 min guided relaxation session incorporating elements of hypnotic induction such as deep breathing and relaxing suggestions (e.g., feelings of warmth, heaviness throughout the body). It is important to clarify that this hypnotic intervention was exclusive to the clinical hypnosis group. The treatment-as-usual group did not receive any form of guided relaxation or hypnotic induction during their assessment; their second phase mirrored the restful, seated position of the first phase, without the introduction of any relaxation techniques or suggestions, to maintain the standard care conditions for this group. The measurement phases followed the recommended experimental structure for HRV studies (29) to assess tonic and phasic HRV. Two individuals separately inspected the ECG files to derive HRV values.

#### Respiration rate

A MindWare respiratory belt (placed below the ribcage) was used to monitor respiration rate during rest and relaxation. Respiration is a potential confounding variable for HRV interpretation, and it is recommended for use as a covariate in HRV analyses if it is found to differ between study groups (29).

#### Heart rate

Heart rate (HR) is a measure of the number of times the heart muscle contracts per minute. HR is an autonomic measure of cardiac output, generally used to index states of psychological stress or relaxation, as well as a pathogenic marker (in the case of elevated HR). The present study assessed HR during rest and relaxation using the MindWare ECG recording.

#### Subjective relaxation

Subjective relaxation was measured using an 11-point numerical scale specifically developed for this study with the question; “On a scale with 10 being the most relaxed you can feel, and 0 being not at all relaxed, how would you rate yourself at this moment?”. These ratings were obtained prior to and after the 5 min rest phase, and 10 min relaxation phase.

#### Pain intensity

The Brief Pain Inventory (BPI-SF) is one of the most widely used scales for measuring pain in patients with a variety of chronic pain problems. The BPI-SF is a 16-item, self-report questionnaire that consists of a body diagram that patients use to mark the location of their pain, a question about pain treatments and medications, and one concerning the percentage of relief obtained. The BPI-SF uses an 11-point NRS (0-10) with end points labeled “no pain” and “pain as bad as you can imagine” to measure the intensity/severity of pain at the present time (“now”). Present pain was used in assessments pre- and post-surgical pain in this study. The BPI has been used extensively in a variety of pain conditions and has been shown to have excellent psychometric properties, including validity and reliability in patients with cancer and chronic nonmalignant pain (31).

### Statistical analyses

Linear mixed models (LMM) were chosen for analyses to enable an intention-to-treat analysis approach (32). We used IBM's SPSS Statistics (version 23) to perform a LMM analysis of the effects of treatment Group (treatment-as-usual, clinical hypnosis) and Time (pre-surgery rest, pre-surgery relaxation, 1-month post-surgery rest, 1-month post-surgery relaxation) on HF-HRV. Subject ID was used as a grouping variable for level 2 (i.e., person-level variables) to account for nesting of level 1 units (HF-HRV) within person. As fixed effects, we entered intercept, group, time, and the interaction term of group by time into the model. Time was included as a repeated factor nested within subject and modeled with a first order autoregressive (AR1) covariance-variance matrix. A random intercept nested within subject was also included in the model to account for idiosyncratic variation due to individual differences in HF-HRV. Significant interactions were examined by simple effects tests and Bonferroni-corrected comparisons. *P*-values were obtained by likelihood ratio tests of the full model with the effect in question against the model without the effect in question. Satterthwaite estimations were used for the degree of freedom denominators, as the SPSS MIXED procedure produces non-integer values that vary from analysis to analysis.

Respiration rate was analyzed using LMM analysis following the same structure as the above LMM analysis with HF-HRV as the dependent variable to determine whether to include respiration rate as a covariate in the HRV analysis as differences in respiration rate present potential confounding influences on HRV and vagal activity interpretation (29). Similarly, LMMs for secondary outcomes were also conducted separately for HR, pain intensity, and subjective relaxation rating variables, with unstructured variance-covariance matrices. Effect sizes (Cohen's d) were calculated using observed means and standard deviations. Baseline characteristics (e.g., age, gender, pre*-*operative pain, pre-operative pain intensity) were analyzed by ANOVA and Chi-square tests of likelihood.

## Results

### Participant flow

**Figure 1:**
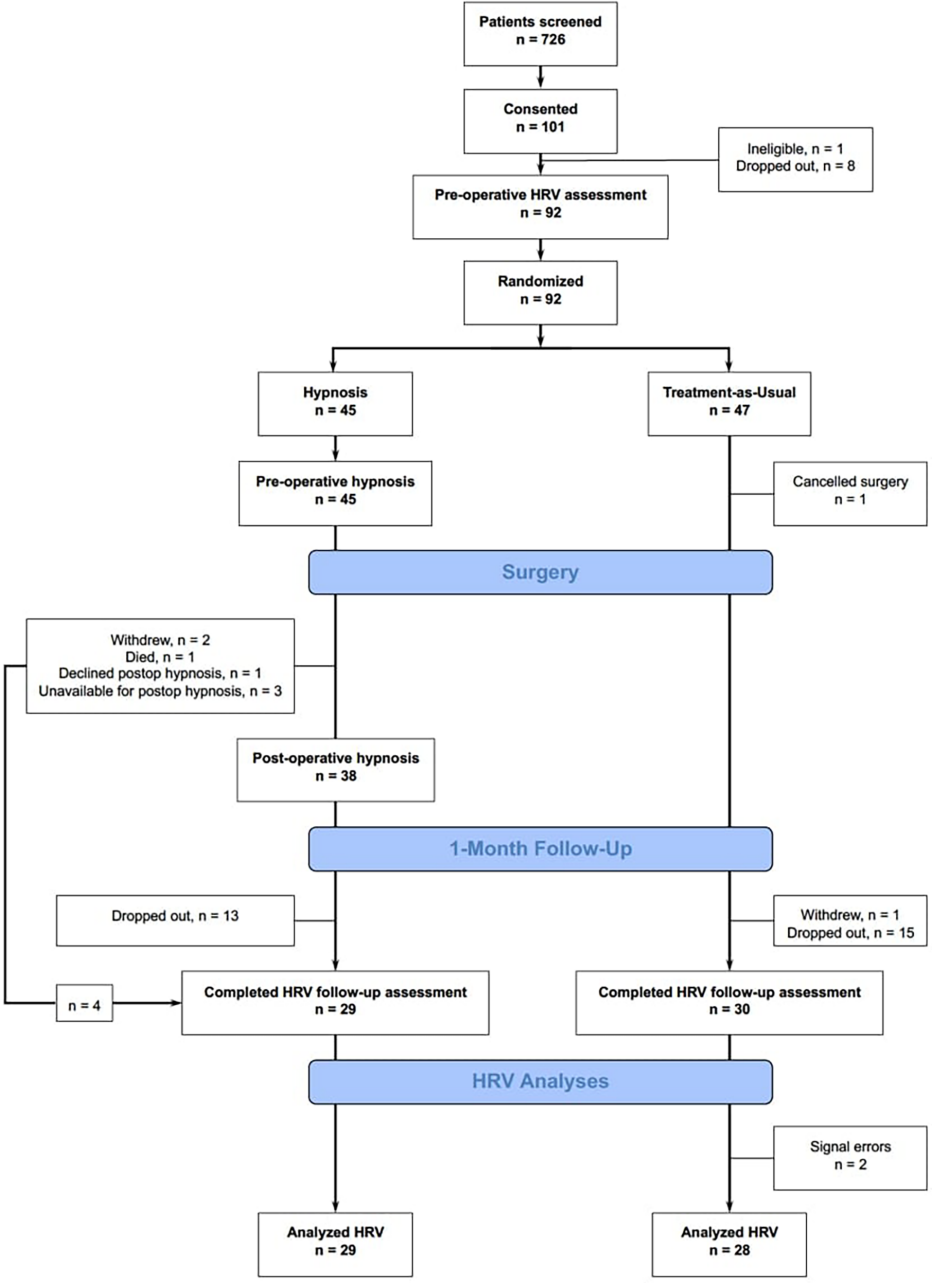
Consolidated standards of reporting trials 2010 flow diagram showing participant flow for enrolment, group classification, randomization to intervention, and analyses. HRV, heart rate variability. All randomized cases were included in linear mixed model analyses, according to an intention-to-treat approach.

Figure 1 shows the CONSORT flow diagram for the present study. The nature of surgical oncology populations often involves complex medical histories and treatment plans, which can limit patient availability and willingness to participate in additional research activities. Concerns about additional time commitments, potential stress associated with participating in research, or simple preference not to participate in research were common reasons for eligible patients declining consent after being pre-screened. Furthermore, logistical challenges, such as scheduling conflicts between the timing of the research activities and the patients' pre-operative preparations or post-operative recovery processes, also contributed to the gap between screened and consented patients. The selection process was designed to balance the need for a representative sample with the practical and ethical considerations inherent in research involving surgical patients. After pre-screening and enrolment, 92 participants were randomized into one of two treatment groups: clinical hypnosis (*n* = 45), and treatment-as-usual (*n* = 47). Nine participants were excluded prior to randomization, and one patient's surgery was cancelled after randomization. All patients in the clinical hypnosis group received a pre-operative hypnosis session at their pre-surgery assessment appointment, and 38 of these patients received post-operative hypnosis. Twenty-nine and 30 patients returned for the 1-month post-surgery assessment appointment in the clinical hypnosis and treatment-as-usual groups, respectively.

### Recruitment

Recruitment commenced on November 6, 2018 and was completed on November 1, 2019 after the number of target participants randomized (*N* = 92) had been reached. Participant attrition rate for the present analyses was 35.6% for the clinical hypnosis group and 36.2% for the treatment-as-usual group. Reasons for dropouts in the clinical hypnosis group included: personal reasons (*n* = 2), death (*n* = 1), unable to schedule follow-up visit (*n* = 5), lost to follow-up (*n* = 8), and developed cardiac complications (*n* = 1). Reasons for dropouts in the treatment-as-usual group included: surgery cancelled (*n* = 1), personal reasons (*n* = 1), unable to schedule follow-up visit (*n* = 1), and lost to follow-up (*n* = 13). Consistent with the intention-to-treat approach, our LMM included all available participant data and preserved missing repeated measures data from a listwise deletion (33). Table 2 shows the number of days between the pre-surgery assessment visit, surgery day, inpatient visits, and 1-month assessment visit for the two groups.

### Data preparation

Two ECG recordings of participants in the treatment-as-usual group could not be analyzed due to insufficient signal. High frequency HRV (HF-HRV) data was log-transformed to reduce skewness (0.60) to acceptable levels (0.27), based on published recommendations (29). Visual inspection of residual plots did not reveal any obvious deviations from homoscedasticity or normality.

Groups did not significantly differ on age, gender distribution, and types of surgeries undergone, as well as pre-existing chronic pain conditions, pain intensity ratings, and prescription pain medications prior to surgery (Table 1).

**Table 1:** Demographic and clinical characteristics of study groups.

Measure	Group	
	Treatment-as-Usual	Clinical Hypnosis
Age in years, M (SD)	55.75 (9.7)	55.93 (12.03)
Gender, *n* %		
Female	16 (57.1)	15 (51.7)
Male	12 (42.9)	14 (48.3)
Surgery type, *n*		
Head & neck	4	3
Liver surgery	4	3
Kidney surgery	1	3
Hysterectomy	5	5
Breast surgery	4	2
Thoracic	3	3
Urologic	3	4
Major Abdominal	3	4
Laparoscopic Abdominal	1	2
Pre-Op Chronic Pain		
Yes	16 (57.1)	14 (48.3)
No	12 (42.9)	15 (51.7)
Pre-Op Present Pain “Now”		
Mean	1.68 (2.29)	1.00
Median	1	0
Range	0–8	0–7
Pre-Op Chronic Pain Condition		
Cancer	2	1
Neuropathic	3	2
Headache	2	2
Orofacial	1	0
Visceral	0	1
Musculoskeletal	13	12
Deep Vein Thrombosis	0	1
Pre-Op Pain Meds		
Yes	15 (53.6)	11 (37.9)
No	13 (46.4)	18 (62.1)
Class of Pre-Op Pain Meds		
Tramadol	0	1
Acetaminophen	9	8
Ibuprofen	4	3
Gabapentin	2	1
Pregabalin	2	1
Topical agents	1	0
Pre-Op Mental Health History		
Yes	1	1
No	27	28
Pre-Op Mental Health Meds		
SSRI	3	1
SNRI	1	0
Anxiolytics	1	0
Stimulants	1	0

**Table 2:** Summary of days between key study timepoints.

	Treatment-as-Usual	Clinical Hypnosis
Pre-surgery assessment and surgery, M (SD)		
Mean # of days between	13.69 (20.12)	8.93 (12.12)
Median # of days between	7	6
Range of # of days between	1–101	1–63
Surgery day and inpatient visit, M (SD)		
Mean # of days between	1.69 (1.11)	1.62 (0.94)
Median # of days between	1	1
Range of # of days between	0–4	1–5
Surgery day and follow-up visit, M (SD)		
Mean # of days between	30.55 (7.09)	31.69 (6.72)
Median # of days between	31	31
Range of # of days between	16–45	21–48
Inpatient and follow-up visit, M (SD)		
Mean # of days between	28.96 (7.34)	29.79 (6.92)
Median # of days between	30	28
Range of # of days between	13–43	20–47

LMM analysis examining respiration rate did not show significant effects of Group, Time, or the Group x Time interaction (Table 3). Following recommendations by Houtveen et al. (34), HRV analyses were conducted without including respiration rate as a covariate.

**Table 3:** Observed means and standard deviations of heart rate and respiration rate in study groups.

Pre-Surgery	Heart rate			Respiration rate		
	Clinical hypnosis	Treatment-as-usual	Total	Clinical hypnosis	Treatment-as-usual	Total
Rest	72.06 (11.29)	69.08 (11.18)	70.62 (11.24)	13.14 (4.03)	12.98 (4.62)	13.06 (4.28)
Relaxation	67.68 (10.62)	71.26 (11.61)	69.50 (11.09)	13.50 (2.66)	13.10 (3.92)	13.31 (3.30)
1-Month						
Rest	77.07 (10.31)	75.70 (11.40)	76.41 (10.77)	13.70 (5.13)	14.29 (4.39)	13.98 (4.75)
Relaxation	75.50 (10.71)	75.69 (11.16)	75.59 (10.83)	13.59 (3.34)	14.18 (4.12)	13.87 (3.71)

With regards to data on frequency of self-hypnosis practice, tracking logs proved to be an unfeasible method to capture this data from patients, as too few patients returned their logs at the 1-month follow-up to enable meaningful analyses of these data. This data analysis was omitted.

### HRV

LMM analysis with Group and Time revealed a significant main effect of Time [*F*(3, 165.77) = 10.32, *p* *< *0.001) that indicated HF-HRV differed across assessments overall. Main effect of Group was not significant [*F*(1, 86.31) = 1.81, *p = *0.18]. In addition, a significant Group by Time interaction effect [*F*(3, 165.77) = 6.58, *p *< 0.001], indicated that HF-HRV change across assessments differed according to treatment group. The interaction effect was examined by simple effects tests using Bonferroni-corrected comparisons regarding Hypotheses 1 and 2 below. HRV data is summarized in Table 3.

#### Hypothesis 1. Higher HRV in clinical hypnosis group than treatment-as-usual group 1-month after surgery

**Figure 2:**
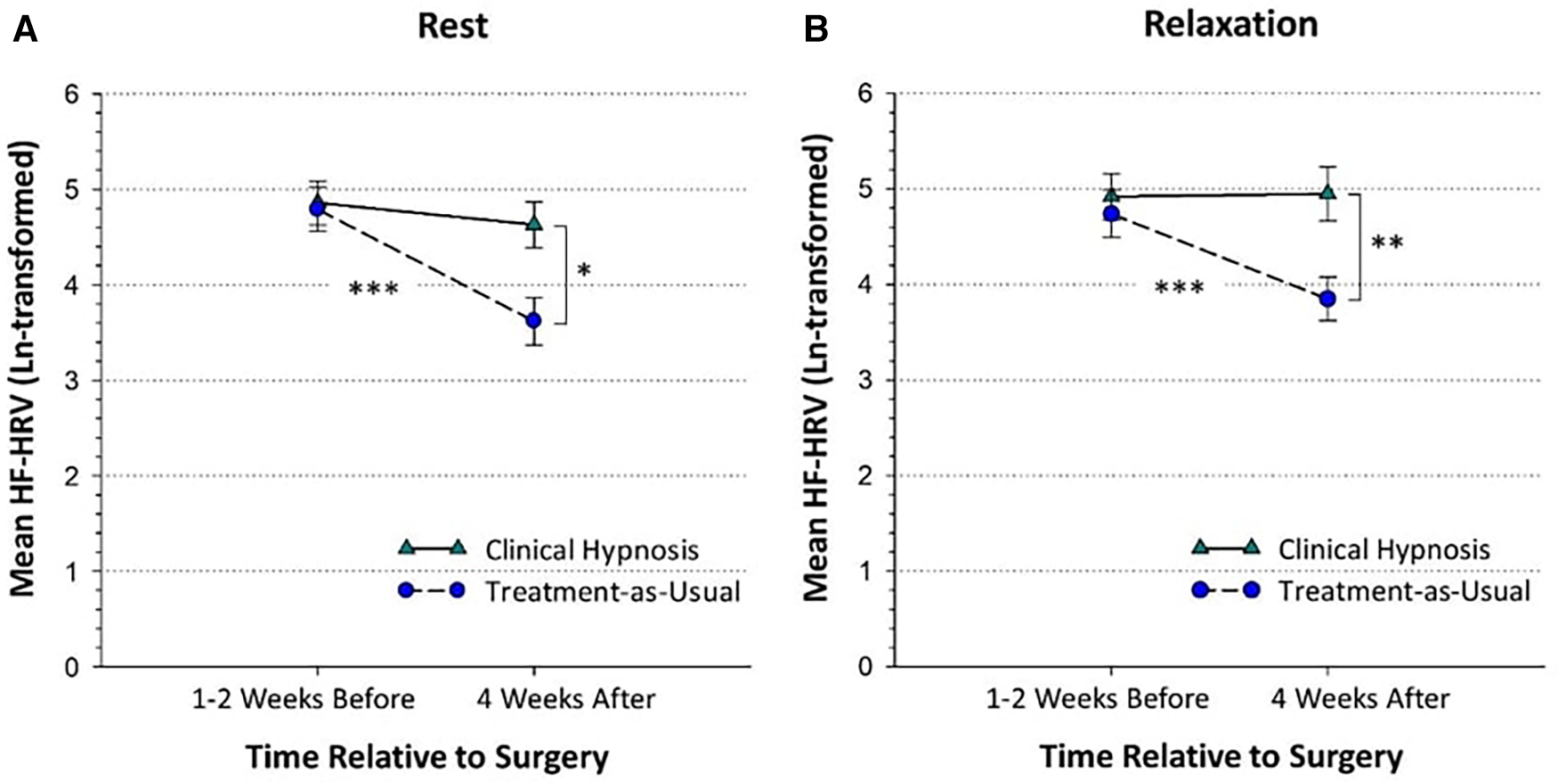
High-frequency heart-rate variability in the clinical hypnosis and treatment-as-usual groups at the pre-surgical and post-surgical assessments shown during rest (panel **A**) and relaxation (panel **B**). The pre-surgical assessment took place before randomization with a mean number of days before surgery of 11.17 (SD = 16.82). The 1-month post-surgical assessment took place a mean of 31.19 (SD = 6.94) days after surgery. HF-HRV = High-frequency heart-rate variability (power as m^2^). Plots are based on observed means and standard errors. *p < 0.05; **p < 0.01; ***p < 0.001.

The simple effect of Group was significant at 1-month for both rest, *F*(1, 171.43) = 5.82, *p *< 0.05, *d *= 0.73 and relaxation, *F*(1, 170.61) = 7.20, *p *< 0.01, *d *= 0.73, indicating that at 1-month after surgery, HF-HRV was higher in the clinical hypnosis group than treatment-as-usual group during both rest and relaxation (Table 4; Figure 2B).

**Table 4:** Observed means and standard deviations of log-transformed high frequency heart rate variability in study groups.

Pre-Surgery	Treatment-as-usual	Clinical hypnosis	Total
Rest	4.74 (1.36)	4.92 (1.29)	4.83 (1.32)
Relaxation	4.79 (1.25)	4.86 (1.53)	4.82 (1.39)
1-Month Post-Surgery			
Rest	3.62 (1.29)^a^	4.63 (1.47)^b^	4.13 (1.46)
Relaxation	3.85 (1.49)^a^	4.95 (1.54)^b^	4.41 (1.60)

^a^ Within-person change compared to pre-surgery measures (*p *< 0.001).

^b^ Between-group difference (*p* < 0.01).

#### Hypothesis 2. Lower HRV in treatment-as-usual group after surgery than before surgery

The simple effect of Time was significant for the treatment-as-usual group, *F*(3,150.70) = 15.67, *p *< 0.001, but not the clinical hypnosis group, *F*(3, 147.60) = 1.05, *p = *0.37, indicating that in the treatment-as-usual group, 1-month rest (*d *= 0.60, *p *< 0.001) and 1-month relaxation (*d *= 0.48, *p *< 0.001) HF-HRV were lower than the corresponding pre-surgery rest and relaxation values (Table 4; Figure 2B) but for the clinical hypnosis group there was no significant change in HRV (rest or relaxation) from pre-surgery to one month post-surgery.

### Heart rate

LMM analysis with Group and Time revealed a significant main effect of Time [*F*(3, 69.47) = 9.87, *p *< 0.001]. Pre-surgery relaxation heart rate was lower than pre-surgery rest heart rate (*p *< 0.01, *d *= 0.06) in all participants. One-month rest and relaxation heart rate were significantly higher than the corresponding values for pre-surgery rest and relaxation heart rate (all *p *< 0.005, all *d *> 0.37). Group and Group × Time interaction effects were not significant.

### Subjective relaxation ratings

LMM analysis with Group and Time indicated a significant main effect of Time [*F*(5, 54.27) = 69.74, *p *< 0.001]. Subjective relaxation ratings obtained after relaxation in the pre-surgery assessment (M = 8.61, SD = 1.88) and at 1-month follow-up (M = 8.66, SD = 1.12) were significantly higher than corresponding pre-surgery pre-rest (M = 6.31, SD = 1.85) and post-rest (M = 7.25, SD = 1.90) ratings, and 1-month pre-rest (M = 6.27, SD = 1.88) and post-rest (M = 7.49, SD = 1.77) ratings in all participants (all *p *< 0.005, *d *> 0.60). The main effect of Group and the Group × Time interaction effect were not significant.

### Pain intensity ratings

LMM analysis with Group and Time (pre-surgery, 1-week post-surgery, 1-month post-surgery) indicated a significant main effect of Time [*F*(2,74.69) = 30.79, *p *< 0.001]. Pain intensity ratings at 1-week (M = 2.93, SD = 2.44) and 1-month (M = 2.20, SD = 2.42) post-surgery were significantly higher than pre-surgery (M = 1.30, SD = 2.14) (*p *≤ 0.001, *d *= 0.28-0.50), and 1-month ratings were significantly lower than 1-week ratings (*p *< 0.005, *d *= 0.21). The main effect of Group and the Group × Time interaction effect were not significant.

## Discussion

The results of the present study show that HRV was significantly higher in the clinical hypnosis group compared to the treatment-as-usual group at the 1-month rest and relaxation time points. Moreover, as hypothesized, HRV significantly decreased from pre- to post-surgical rest and relaxation assessments in the treatment-as-usual group but not the clinical hypnosis group.

### Effects of clinical hypnosis on 1-month post-surgical HRV

The central implication of the present findings is the demonstrated potential for peri-operative clinical hypnosis to impart a protective effect on PNS functioning. The results suggest that clinical hypnosis prevents the deleterious effects of surgery on HRV, by helping to preserve pre-operative levels of PNS functioning, in contrast to the treatment-as-usual group that displayed an expected course of HRV reduction in the absence of clinical hypnosis and in the context of post-surgical pain and inflammation. Future studies are needed to better understand the acute effect of hypnosis on vagal HR modulation, specifically studies featuring assessment of HRV during peri-operative clinical hypnosis sessions, and during the first several post-operative days after patients receive peri-operative hypnosis sessions. Further investigations might also include measurement of other downstream effects of vagal activity, such as pro-inflammatory cytokines.

Study results suggest major surgery results in persistent withdrawal of vagal HR modulation activity up to 1-month post-surgery in participants assigned to the treatment-as-usual group but not the clinical hypnosis group. This finding corroborates and extends previous literature reporting significant HRV reductions in patients in the days and weeks after major surgery (8–10, 35). Major surgery comprises acutely stressful procedures in which pain is elicited due to tissue damage, inflammation, and central sensitization (12) all of which can have deleterious effects on vagal-mediated HRV (23). Hildenborg et al. (36) recently found that pre-operative HRV associates with differential inflammatory response patterns and observed comparatively elevated HRV during the weeks following surgery in the high (vs. low) HRV group. The authors postulated that healthy, flexible HRV response patterns associate more with a timelier restoration of inflammatory homeostasis than lower, static HRV. Hence, promoting vagal HR modulation in patients before and after planned surgeries may be a feasible adjunct to improving postoperative outcomes. Theoretically, preserved PNS functioning can augment post-surgical recovery via the vagal anti-inflammatory reflex (17) and thereby inhibit inflammatory activity that increases risk of persistent post-surgical pain.

The results from the treatment as usual group show that some degree of diminishment in PNS activity occurred at the one-month time point. Adequate post-surgery recovery and healing is likely marked by a gradual restoration of PNS functioning. However, we do not know the pattern of HRV activity in the days after surgery in the clinical hypnosis group. It would be important to establish the trajectory of HRV recovery after surgery (13). It is possible that peri-operative hypnosis positively alters a patient's trajectory in terms of coping with the aftermath of surgery by entraining them with a vagal-activating practice before and after surgery. Future RCTs may yield more insight into the ebb and flow of sympathetic and vagal HR modulation post-surgery via ambulatory HRV monitoring throughout the day to elucidate the process of PNS restoration post-surgery. In this regard, investigation of hypnosis as an adjunct in post-surgical populations at elevated risk for complications of post-surgical autonomic activation (i.e., vascular surgery patients, cardiac surgery patients) would be a promising future direction to explore.

One of the more puzzling results of the present study is that, in contrast to the HRV findings, we did not observe a similar group by time (pre- or 1-month post-surgery) interaction for subjective relaxation and pain ratings. This makes it somewhat challenging to interpret the HRV results, because the clinical hypnosis intervention was not only designed to impact post-surgical HRV, but to leverage its theoretical PNS benefits to enhance post-surgical relaxation and pain relief. However, resting state HRV is a general marker of the responsiveness and flexibility of the ANS (27), and is not necessarily a direct indicator of a person's state relaxation and current pain levels. In addition, HRV during relaxation assesses the capacity to increase cardiac vagal control (37), and similarly, may not be readily associated with downstream, self-reported variables such as state relaxation and pain. It is likely that the effects of hypnosis on HRV are similar to other physiological parameters (e.g., blood pressure, gut motility) in that they are not directly accessible to psychological introspection and self-report [see (38)].

The short and long-term impact of peri-operative clinical hypnosis on HRV may be understood by the effects of hypnotic suggestions that promote deep physiological relaxation and breathing techniques that assist with pain coping (5). Patients guided into deeply relaxed physiological states and primed with positive expectations via hypnosis may be better equipped to have a gradual and efficient restoration of PNS functioning post-surgery. In the current study, a relaxation response was induced through deep breathing and imagery-based suggestions during both pre- and post-surgery hypnosis sessions. These hypnosis sessions aimed to reduce pain and induce relaxation through adaptive and gentle dissociation from pain awareness and imaginal superimposition of pleasurable sensations onto body parts (5). Both relaxation and top-down pain regulation are characterized by reduced sympathetic and increased parasympathetic control of the ANS, reflected in higher HRV (39).

### Effects of clinical hypnosis on subjective relaxation

It is noteworthy that HRV did not increase significantly after the relaxation task during assessments, in which patients were provided audio guidance of a 10 min practice including deep breathing and relaxing suggestions (e.g., warmth, heaviness throughout body). These instructions were also used by clinicians during the hypnotic induction stage of the pre- and post-surgery clinical hypnosis sessions provided to the clinical hypnosis group. Interestingly, 1-month relaxation HRV appears to trend upward from corresponding rest HRV values in the clinical hypnosis group, however, a significant HRV increase was not detected. Perhaps there is an underlying dose effect, and a duration of more than 10 min of relaxation is needed to capture a significant HRV increase associated with relaxation in the clinical hypnosis group. Alternatively, if pre-surgical clinical hypnosis helps preserve HRV, one possibility is that it might not be predominantly attributed to the relaxation effect of the hypnotic induction states. As such, the latter and deeper stages of hypnotic dissociation and positive expectancy suggestions may play an integral role in promoting vagal HR modulation. By contrast, subjective relaxation ratings showed a pattern of significant increases from pre-rest, post-rest, to post-relaxation similarly across both pre-surgical and 1-month post-surgical assessments. Having received perioperative clinical hypnosis was not associated with reporting higher subjective relaxation values at 1-month post-surgery. While higher HRV would be expected to be positively associated with subjective relaxation, this could largely depend on idiosyncratic variations in interoception (i.e., ability to accurately monitor one's internal bodily states). Furthermore, it may be that longer and deeper states of hypnosis (i.e., dissociation) may be required for the dynamics of subjective and physiologically relaxed states to coalesce.

Patients were encouraged to maintain a self-hypnosis practice by using audio recordings (accessible via a YouTube channel) before and after surgery. Self-hypnosis practice may have contributed to a cumulative restorative effect on vagal-mediated HRV in the clinical hypnosis group, explaining the differences observed at 1-month. Future studies should consider using more feasible procedures (e.g., phone/web-based surveys, or automatic tracking through a digital app) to monitor self-hypnosis practices in order to elucidate the effective “dose” in the surgical context.

### Effects of clinical hypnosis on heart rate and pain

Analyses of physiological data revealed that all patients exhibited significantly elevated HR during rest and relaxation assessments at 1-month post-surgery. Net ANS activity shifted towards greater sympathetic activity for all patients post-surgically. One factor to explain this would be the significantly higher post-surgical pain ratings at one-month, since pain and sympathetic activity are interconnected functions (23). Pain is generally associated with “sympathetically dominant” states (e.g., high HR, lower HRV), making the preservation of parasympathetic tone vital to stress and pain regulation after surgery. Another potential reason for the HR elevations may be that they signal the relative decline in fitness level of patients, due to the physical deconditioning that accompanies post-surgical recovery, when patients are engaging in less physical activity. While changes in pain intensity ratings post-surgery were found, the differences between the CH and TAU groups did not reach the threshold of 1.39 points that would indicate a clinically significant difference (40), It will be important for future studies to conduct a more nuanced investigation of the effects of peri-operative hypnosis on post-surgical pain, including variables such as individual pain thresholds, psychological factors, and surgical factors.

### Limitations and future directions

The current study has limitations. Firstly, this study was not double-blind. To keep risk of bias as low as possible, treatment allocation concealment was used so it was unknown to study staff which patients would be randomized to clinical hypnosis or treatment-as-usual arms until pre-surgical assessments had been completed. Furthermore, this study lacked an active control condition. The lack of adequate “sham” or placebo hypnosis procedures to enable appropriate controls for clinical hypnosis (28) poses challenges in designing active control conditions in hypnosis research. We decided against employing sham or placebo control conditions for ethical reasons. It is worth noting that the TAU group received a brief relaxation procedure prior to surgery, as part of the pre-surgery HRV assessment, which is more than what a typical control group receives in perioperative studies. A suitable control intervention might include peri-operative sessions of relaxation (e.g., deep breathing). Other important factors to investigate in future studies include sleep and surgery type.

It is important to highlight that a possible confounding variable in this study was participants' use of an unaccounted amount of self-hypnosis practice. Future studies are recommended to have participants monitor their self-hypnosis practice (e.g., duration and frequency) to enable analyses of potential dose-response curve effects. Additionally, the relationships between HRV and opioid consumption in patients receiving peri-operative hypnosis warrants further investigation with possibly larger samples or different methodological approaches to fully understand these complex relationships. Finally, future studies may consider examining other validated HRV metrics (e.g., low frequency, root mean squared of successive differences) to yield further insights.

Taken together, the findings of the main RCT study and the present secondary study have shown that perioperative clinical hypnosis has opioid-sparing effects and protective effects on pain catastrophizing and vagal-mediated parasympathetic function considered to play a key role in post-surgical recovery. It is hoped that future studies can further build on these findings in demonstrating the effectiveness of perioperative clinical hypnosis as an adjunct treatment in the surgical setting.

## Data Availability

The datasets presented in this article are not readily available because data can't be shared. Requests to access the datasets should be directed to jkatz@yorku.ca.
